# The Influence of Fermenting Yeast on the Sensory Properties of *Graševina* Wine

**DOI:** 10.3390/foods10112752

**Published:** 2021-11-10

**Authors:** Igor Deak, Kristina Habschied, Josip Mesić, Jurislav Babić, Dragan Kovačević, Viktor Nedović, Krešimir Mastanjević

**Affiliations:** 1 Studia Superiora Posegana, Polytechnic in Požega, 34000 Požega, Croatia; igordeak30@gmail.com (I.D.); jmesic@vup.hr (J.M.); 2 Faculty of Food Technology Osijek, Josip Juraj Strossmayer University of Osijek, F. Kuhača 20, 31000 Osijek, Croatia; jbabic@ptfos.hr (J.B.); dragan.kovacevic@ptfos.hr (D.K.); kmastanj@gmail.com (K.M.); 3 Faculty of Agriculture, University of Belgrade, 11080 Zemun, Serbia; viktor.nedovic@mpn.gov.rs

**Keywords:** *S. cerevisiae*, *T. delbrueckii*, white wine, non-*Saccharomyces*, fermentation

## Abstract

Recent research has showed a breakthrough in investigating the effect of non-*Saccharomyces* yeast on wine quality and sensory properties. The aim of this study was to compare the influence of conventional yeast, *Saccharomyces cerevisiae*, vs. that of the non-*Saccharomyces* *Torulaspora* *delbrueckii* on the sensory profile of the white wine *Graševina*, and to establish if there are any differences in physical–chemical properties in regards to the applied yeast. Sample One was inoculated with both yeasts, while Sample Two was inoculated only with *S. cerevisiae*. The results indicated that a combination of *T.* *delbrueckii* and *S. cerevisiae* resulted in somewhat higher ethanol content in the finished wine. Sensory evaluation showed no significant discrepancies for any of the wines. Aspect and flavor were graded similarly, but the quality and intensity of the bouquet of Sample One was graded somewhat higher (14 and 6.6) than Sample Two (13.6 and 6.4). These findings open a very wide gate for future research in white wines.

## 1. Introduction

The use of non-*Saccharomyces* yeast is becoming more and more popular, even though for many years they had been considered contaminants. However, due to their positive effects on wine aroma, the utilization of such yeast has become preferable [1]. They tend to accelerate the initial stages of a wild ferment, and they are actually more pronounced in the vineyard than *Saccharomyces cerevisiae* [2]. Overall wine properties, especially aroma, is greatly correlated with the enzymatic profile of yeasts; semi-fermentative yeast genera (*Hanseniaspora* spp., *Metschnikowia* and *Candida* spp.) tend to display a somewhat weaker tolerance to ethanol which can be restrictive during the later stages of fermentation [3,4]. There are non-*Saccharomyces* yeasts that can endure high ethanol concentrations and influence the aroma profile of wine [5].

One commercially available yeast that is recognized for its ability to actively withstand the later stages of indigenous ferments is *Torulaspora delbrueckii* [6]. Yeast belonging to the *Torulaspora* genera are highly fermentative, tolerant to alcohol, and have good fermentation rates in wine as they tend to dominate in the later stages of fermentation [3,6,7]. As *Torulaspora delbrueckii* displayed almost parallel fermentative characteristics to *S. cerevisiae*, it became the first commercially available non-*Saccharomyces* yeast for wine fermentation [8]. Certain setbacks came into focus in the wineries contaminated with *S. cerevisiae* strains which enabled *T. delbrueckii* to proliferate and prevail over the *S. cerevisiae* [9]. To overcome this obstacle, killer strains of *T. delbrueckii* that successfully inhibited the growth of *S. cerevisiae* were developed [10]. By reducing the concentrations of common ethyl esters and increasing the concentration of lactones and lesser-known esters, these strains contribute to the complexity of wine aroma [11] by adding dried fruit/pastry aromas instead of fresh fruity aromas. *T. delbrueckii* shows a better performance in oxygen-deprived conditions, which makes it more suitable for the fermentation of red wines; this is because, during the fermentation of red wines, a certain amount of oxygen is added to the ferment when breaking up the skin cap. White and sparkling ferments are fermented under strictly anaerobic conditions [12]. However, to test the effect of *T. delbrueckii* on white wines, we fermented grape cultivar *Graševina* (*Vitis vinifera* L.) with the addition of this yeast. Wine production is significant economy branch in the Republic of Croatia and the largest share of grapes belong to *Graševina* variety [13]. *Graševina* is a common wine variety in Croatia and neighboring countries such as Hungary, Austria, and Serbia, to an extent. The *Graševina* variety produces particularly valued “muddy wines” (sand wines) [14].

Even though all Croatian regions are considered suitable for grape growing, the Danube region has by far the most potential for growing different grape wine varieties cultivated in mildly continental climate. The largest parts of the Danube region are considered to be ecologically suitable for grape wine cultivation. There are three major vineyards in this part of Croatia—Srijem, Erdut, and Baranja.

In the last 10 years, a lot of effort and attention has been invested in the selection of yeast varieties and their role in wine with regard to aromatic expression, as well as to emphasize the particular style of wine, with respect to the typical climate [14].

The aim of this research is to determine the influence of the conventional yeast genus *Saccharomyces* (species: *S. cerevisiae*) and the non-conventional yeast genus *Torulaspora* (species: *T. delbrueckii*) on the sensory profile of *Graševina* wine in real winery conditions. The differences in physical–chemical parameters, with respect to the applied genus and type of yeast, were also evaluated. The research work was carried out on *Graševina* variety grapes harvested in the Josić winery in Zmajevac.

## 2. Materials and Methods

### 2.1. Preparing the Must and Fermentation

After picking, the total amount of grapes was 8.950 kg. The primary process, the mashing of grapes (crushing and destemming) was performed with the addition of 7 g of K_2_S_2_O_5_ per 100 kg of grapes. Pressing was carried out by pneumatic press. During pressuring 3 mL of ENDOZYM^®^ E-FLOT (AEB, San Polo, Italy) was added per hL of must. This enabled the pectinase to break down more quickly, allowing a better rise of the flock during the flotation stage. The next day, must flotation was carried out with the addition of 20 mL of E-GEL (AEB, San Polo, Italy) per hL of must. The following actions were undertaken:

- the must was left for 3 h to clarify;
- after 3 h the clarified must was decanted into two tanks with a volume of 2630 L;
- each tank was inoculated with the selected yeasts (Sample One and Sample Two).

The starting must had the following basic composition (Table 1):

### 2.2. Vinification and Fermentation

Sample One consisted of clear and decanted must inoculated with the selected enological yeast *Torulaspora delbrueckii* (BIODIVA^®^, Lallemand, Montreal, QC, Canada) (20 g/hL). *T. delbrueckii* is known to contribute an aromatic complexity to wine. In order to achieve successful propagation and fermentation, prior the addition of yeast the water, in which 15 g/hL GO- FERM PROTECT^®^ (Lallemand, Montreal, QC, Canada) was added, was set to 35 °C. The must temperature during inoculation was 15 °C. After a week, a significant decrease of specific gravity was noted (11 °Oe). At this stage, to ensure the complete fermentation, the must was further inoculated with 25 g/hL of selected wine yeast *Saccharomyces cerevisiae*. This yeast was prepared similarly, with the addition of 15 g/hL GO-FERM PROTECT^®^ (Lallemand, Montreal, QC, Canada). Additionally, the must was fortified with 10 g/hL FERMAID^®^ E (Lallemand, Montreal, QC, Canada) to ensure faster fermentation and prevent a possible halt. *Torulaspora delbrueckii* could be detected in the must at the very beginning of fermentation, and when the specific gravity of the must fell by 10 to 15 degrees, *Saccharomyces cerevisiae* acted detrimentally to *Torulaspora delbrueckii* cells and prevailed. By associating non-conventional yeast with *Saccharomyces cerevisiae*, fermentation results in a desirable paste, and the non-*Saccharomyces* yeast contributes to the aroma.

Sample Two was prepared in a similar way, by adding 7.5 g/hL FERMOL^®^ AROME PLUS (*Saccharomyces cerevisiae*) (AEB, San Polo, Italy) and 7.5 g/hL FERMOL^®^ CRYOAROME (*Saccharomyces cerevisiae*) (AEB, San Polo, Italy) into the clear and decanted must. The inoculation temperature of the must was 15 °C. After the inoculation, temperature was set at 12–13 °C.

For both wines, the fermentation was finished after a month, and the tanks were filled to the top. First racking was carried out after 2 months, with the addition of 35 mL/hL of 5% H_2_SO_3_. Six months after the inoculation, the clarification of the wine with the addition of bentonite and gelatin was performed. After another month, the wine was filtered and was ready to consume in June, eight months after the inoculation.

### 2.3. Analysis and Sensory Evaluation

The wine samples used for physical–chemical analysis were excluded from wine tanks during a period of aging and storing. Sensory evaluation was performed in the vinery Josić. The method included up to 100 positive points. Evaluation was conducted by five evaluators certified by Croatian Centre for Viticulture, Enology and Edible Oils Analysis.

#### 2.3.1. Physical–Chemical Analysis

Sugar concentration was determined by Oechsel scale (°Oe).

Concentration of unfermented sugars in the must was determined using a refractometer (°Oe).

Total acids in must and wine were determined by titration with 0.1 M NaOH, with bromothymol blue as indicator.

Free SO_2_ in must was determined according to Ripper [15].

The pH of must and wine was measured portable pH meter (Mettler Toledo, Columbus, OH, USA).

Ethanol was determined according to Salleron, using an electric ebulliometer (Enartis, Winsdor, CA, USA) [16].

Volatile acids were analyzed using a semimicro distillation method described by (Weinlabor Schliessmann, Schwäbisch Hall, Germany).

#### 2.3.2. Sensory Evaluation

Sensory evaluation was conducted in a well-lit and ventilated room, in the absence of noise and smells. Room temperature was between 20 °C and humidity ranged from 60 to 70%. Evaluators had the opportunity to rinse their glasses, pour the sample and to neutralize their taste buds. Glasses were appropriate for the selected wine and samples were served at the prescribed temperature for white wines, 10–12 °C. Sensory evaluation was conducted by a 100-point scale. The final grade was obtained by calculating the arithmetic mean (after the highest and the lowest grade had been discarded). A minimum of 50% of the evaluators had to confirm that the wine had a distinction that had been enhanced [14].

## 3. Results and Discussion

There are number of reports about non-*Saccharomyces* yeasts in wine making, but there is still not enough information to draw a rectilinear conclusion on their impact on wine quality. This is mostly caused by the fact that many of these studies were conducted in a controlled environment (e.g., laboratory-scale trials carried out on synthetic media or sterilized grape musts), which greatly differs from the conditions commonly encountered in a winery [17,18]. However, since the popularity of non-*Saccharomyces* yeast is rising and it is becoming more interesting for winemakers, it is important to test as many of these strains as possible prior to their commercial availability. The best way to do so is to test them in real winery conditions [18].

Table 2 shows minor differences in temperature at the very beginning of fermentation, although both musts were of identical temperature upon inoculation. We also noticed that, according to the amount of unfermented sugars, Sample Two started the conversion of sugar somewhat faster, leading to a faster fermentation course. Towards the end of fermentation, we noticed significant differences in the fermentation temperature of the must, which was 2–2.5 °C lower in the second sample than in the first sample. At the end of the fermentation, the content of unfermented sugars was almost exactly the same in both samples, which indicates that both yeasts were successful in attenuating sugars.

In Table 3 (wines were analyzed 1.5 and 4 months after inoculation) it can be seen that the amounts of free sulfur, volatile acids, and total acids were approximately the same in both samples, and that there was also a minimal difference in the pH values of the wine. This indicates that none of the yeasts used stood out in their activity in terms of these parameters. There was, however, a significant difference in the amount of produced alcohol during the first month and a half into fermentation. In Sample One, there is a 0.4% (*v*/*v*) higher ethanol content than in Sample Two, which is not negligible. Free sulfur increased over time in both samples, and was approximately 14% higher in Sample One after 160 days of fermentation. Sample Two showed a similar increase, 25% higher, after 160 days of fermentation. This could be attributed to the fact that yeast strains produce SO_2_ during fermentation [19].

The results of the analysis performed 4 months after inoculation show that Sample One contained a slightly (but statistically significant) lower total acid content than Sample Two. Sample Two showed a significantly higher content of volatile acids, which could have presented a problem if they had increased too much. At this stage of production, however, the volatile acid content appeared to be within the normal range. According to several authors [20,21,22], a mixed inoculation of *T. delbrueckii* and *S. cerevisiae* act to reduce off-flavor compounds such as volatile acidity, acetaldehyde, and acetoin, which also seems to be the case in our research. Total acidity showed no significant difference between samples, even after four months into fermentation, as did pH values. Ethanol levels stayed the same as in the first analysis and displayed no significant difference between samples.

**Figure 1:**
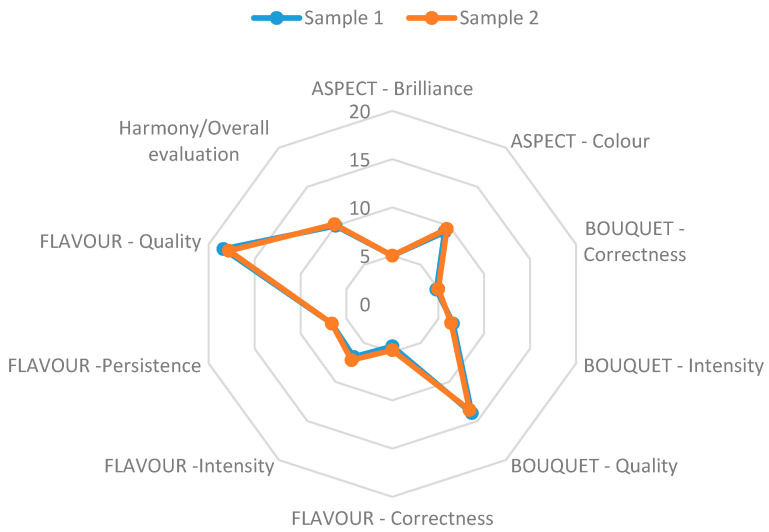
Results of sensory analysis of both samples.

Figure 1 shows the results of the sensory analysis of the produced wines. The results indicated that no significant difference was noted between the samples, but there were slight differences in total scores for color; Sample Two scored 9.6, while Sample One scored 9.2. This indicated that *T. delbrueckii* had a certain effect on the color of *Graševina* wine and further analysis is required in order to understand the complexity of this phenomenon. Brilliance was evaluated equally (5.0). Correctness of bouquet was also evaluated and the results were in favor of Sample Two again; the evaluators gave a slightly higher grade to Sample Two (5.0) in comparison to Sample One (4.8). Sample One received higher grades for the intensity of its bouquet (6.6) and its quality (14.0).

The flavor of wine has several properties: correctness; intensity; persistence; and quality. Correctness was evaluated as being higher in Sample Two, 4.8, vs. Sample One with 4.4. A significantly lower grade was appointed to Sample One for its intensity of flavor (6.8), while Sample Two received 7.2 in this property. Persistence was evaluated as being equal in both samples (6.6), but quality of flavor was significantly higher in Sample One (18.4). Sample Two was graded with 17.8.

Harmony and overall evaluation were better for Sample Two, with scores of 10.2 and 86, while Sample One received 10.0 and 85.6. The differences are small, but indicate that standard production with *S. cerevisiae* is still preferable. This might be because *T. delbrueckii* produced or emphasized certain flavors that were non-typical for this particular wine variety.

## 4. Conclusions

The rapid onset of the fermenting process is one of the most important properties of yeast when employed in alcoholic fermentation. It is very important for the beginning of alcoholic fermentation that the inoculated yeast adapts as quickly as possible to new and possibly unfavorable environmental conditions (temperature, etc.). This ensures a quick and even fermentation process. The research shows that yeast from the genus *Saccharomyces* adapted better in the *Graševina* must than yeast from the genus *Torulaspora*. This was expected, since the yeasts from the genus *Torulaspora* exhibit less fermentation activity than the genus *Saccharomyces*. The obtained results show that fermentation conducted with a combination of yeasts from the genus *Torulaspora* and *Saccharomyces* resulted in a slightly higher alcohol content in the wine sample, whilst wine where only the genus *Saccharomyces* was used had a lower alcohol content. This could be due to the metabolic differences (i.e., resistance to ethanol content, fermentation/attenuation of sugars, etc.) between the genera. From the performed wine analyses, we conclude that the yeast from the genus *Saccharomyces* produced a significantly higher proportion of volatile acids, expressed as acetic acid. For wine yeasts, it is more desirable to produce as few volatile acids as possible during fermentation, as this later affects the quality of the wine itself.

The sensory evaluation of the wine concluded that there were no significant differences in color and clarity; the studied genera of yeasts had no significant effect on wine appearance, but a very small advantage in points was given to the sample from the genus *Saccharomyces*. Regarding the evaluation of wine taste, both studied samples were very similar in terms of average evaluation, and it is difficult to conclude from such minimal differences whether one of the studied genera was superior to the other. A more significant difference appeared in the quality of the taste, which was better evaluated in the sample fermented with combined yeasts, Sample One. The most interesting differences were recognized in bouquet evaluation, where the sample fermented with combined yeasts received an on average higher grade than the sample where yeasts only from the genus *Saccharomyces* were employed. This proves the positive effect of combined inoculation on the quality and intensity of *Graševina* wine. It is also worthy of note that only the study of double inoculation by producers of applied commercial yeast is aimed precisely at emphasizing the aromatic complexity in white wines that have low aromatic potential.

In conclusion, yeasts are the most important factors when it comes to alcoholic fermentation significantly affecting the final quality of wine, even when viewed from various perspectives. By isolating and commercializing different genera, species, and strains of yeast, wine producers could create their own style of wine, targeting certain properties of this future wine, and thus put a “signature” on their creation. However, fermentation in real winery conditions is necessary to establish the real influence of any tested yeast on wine quality. Further research should be employed in order to gain a better view on the different fermentation metabolites in wine (flavor, bouquet, smell, and overall quality) originating from both *Saccharomyces* and non-*Saccharomyces* yeast.

## Figures and Tables

**Table 1:** Basic composition of clarified must prior to fermentation.

Component	Amount
Sugars	100 °Oe
Total acids	6.3 g/L
Free SO_2_	11.52 mg/L
pH	3.30

**Table 2:** The temperatures and fermentation course during production of *Graševina* wine.

Time after Inoculation (Days)	Temperature (°C)		Unfermented Sugars (°Oe)	
	Sample One	Sample Two	Sample One	Sample Two
7	15.4	14.6	74	70
8	15.1	14.9	65	50
9	15.1	14.9	55	41
10	15.2	14.7	48	37
13	14.1	13.5	46	34
14	16.0	12.5	37	33
18	15.7	12.1	32	33
21	15.7	12.5	30	32
25	15.6	12.5	30	32

**Table 3:** Basic physical–chemical content of fermenting wine.

Component Results		
**45 Days into Fermentation**		
	Sample One	Sample Two
Free sulphor mg/L	16.64 a ± 0.35	15.36 b ± 0.41
Volatile acids g/L	0.34 a ± 0.08	0.31 a ± 0.09
Total acids g/L	5.91 a ± 0.13	6.05 a ± 0.11
pH	3.39 a ± 0.12	3.32 a ± 0.09
Ethanol %	14.2 a ± 0.25	13.8 a ± 0.31
**160 Days into Fermentation**		
Free sulphor mg/L	19.20 b ± 0.33	20.48 a ± 0.40
Volatile acids g/L	0.37 b ± 0.11	0.54 a ± 0.08
Total acids g/L	5.61 a ± 0.16	5.92 a ± 0.14
pH	3.34 a ± 0.17	3.35 a ± 0.15
Ethanol %	14.2 a ± 0.25	13.8 a ± 0.31

Values shown are the means of two measurements. Values displayed in the same row and tagged with different letters (a,b) are significantly different (*p* < 0.05).

## Data Availability

The data presented in this study are available on request from the corresponding author. The data are not publicly available due to technical reasons.
